# Aggressive behavior as a predictor of home range size: findings from both range-restricted and widespread Darwin’s finch species

**DOI:** 10.1007/s10336-024-02215-7

**Published:** 2024-09-21

**Authors:** Jefferson García-Loor, Mario Gallego-Abenza, Andrew C. Katsis, Verena Puehringer-Sturmayr, Diane Colombelli-Négrel, Çağlar Akçay, Sonia Kleindorfer

**Affiliations:** 1 Department of Behavioral and Cognitive Biology, University of Vienna, 1030 Vienna, Austria; 2 Konrad Lorenz Research Center for Behavior and Cognition, University of Vienna, 4645 Vienna, Austria; 3 Department of Zoology, Stockholm University, Stockholm, Sweden; 4 College of Science and Engineering, Flinders University, Adelaide, 5001 Australia; 5 School of Life Sciences, Anglia Ruskin University, Cambridge, UK; 6 Department of Psychology, Koç University, Istanbul, Turkey

**Keywords:** Behavioral traits, Telemetry, Darwin’s finches, Aggressiveness, Home range

## Abstract

Information about an animal’s home range (the geographical area in which it accesses resources for survival and reproduction) is vital broadly for understanding animal behavior and specifically for developing conservation management plans. Although personality traits—consistent individual differences in behavior across time and contexts—may affect an animal’s home range size, we still lack a breadth of empirical studies across systems to fully appreciate this influence. Here, we tested the relationship between behavioral responses and home range size in two Darwin’s finch species on Floreana Island: the critically endangered and range-restricted Medium Tree Finch (*Camarhynchus pauper*) and the common and widespread Small Ground Finch (*Geospiza fuliginosa*). Using a combination of rapid-assessment assays during both short-term captivity and in the field, we measured exploration and aggressiveness in males from both species. We then used radio telemetry to measure each bird’s home range size over a week-long period. We predicted that (1) fast-exploring individuals would have larger home ranges, and (2) more aggressive individuals would have smaller home ranges. We found that Medium Tree Finches had smaller home ranges than Small Ground Finches, that exploratory behavior was positively correlated with home range size only in Small Ground Finches, and that, in both species, individuals’ aggressiveness was negatively correlated with home range size, whereby the more aggressive individuals occupied smaller home ranges. We conclude that behavioral responses that align with major personality traits can predict home range size, which may provide an important tool for the conservation and management of endangered species when, for example, selecting individuals with different personality profiles for reintroduction.

**Supplementary Information:**

The online version contains supplementary material available at 10.1007/s10336-024-02215-7.

## Abstract (de)

**Aggressivität korreliert mit der Größe des Aktionsraums sowohl bei häufigen als auch bei seltenen Darwinfinkenarten**

Die Kenntnis der Größe des Aktionsraums eines Tieres („home range size“: das Gebiet, das ein Individuum benötigt, um alle überlebensnotwendigen Aktivitäten wie Nahrungssuche, Ruhe und Fortpflanzung auszuführen) ist essenziell für das Verständnis des Tierverhaltens und die Entwicklung von Managementstrategien im Artenschutz. Obwohl Persönlichkeitsmerkmale – konsistente individuelle Unterschiede im Verhalten über Zeit und Kontexte hinweg – die Größe des Aktionsraums beeinflussen können, mangelt es an vergleichenden Studien über mehrere Systeme hinweg. In der vorliegenden Untersuchung wurde der Zusammenhang zwischen Erkundungsverhalten, Aggressivität und Aktionsraumgröße bei zwei Darwinfinkenarten auf der Insel Floreana analysiert: dem vom Aussterben bedrohten Kleinschnabel-Darwinfinken (Camarhynchus pauper) und dem häufigen, weit verbreiteten Kleingrundfinken (Geospiza fuliginosa). Erkundungsverhalten und Aggressivität von Männchen beider Arten wurden durch Schnelltests erfasst, die sowohl in kurzer Haltung als auch im Feld durchgeführt wurden. Die Aktionsraumgröße wurde anschließend mithilfe der Radiotelemetrie über einen Zeitraum von einer Woche bestimmt. Unsere Hypothese war, dass Individuen mit ausgeprägtem Erkundungsverhalten größere Aktionsräume und solche mit höherer Aggressivität kleinere Aktionsräume aufweisen würden. Die Ergebnisse zeigten, dass Kleinschnabel-Darwinfinken kleinere Aktionsräume hatten als Kleingrundfinken. Nur bei den Kleingrundfinken korrelierte das Erkundungsverhalten positiv mit der Aktionsraumgröße. Bei beiden Arten bestand eine negative Korrelation zwischen Aggressivität und Aktionsraumgröße, wobei aggressivere Individuen kleinere Aktionsräume besetzten. Diese Ergebnisse deuten darauf hin, dass Persönlichkeitsmerkmale wichtige Hinweise auf die Größe des Aktionsraums liefern können, was wiederum ein entscheidender Faktor für den Artenschutz sein könnte – insbesondere bei der Auswahl von Individuen mit unterschiedlichen Persönlichkeitsprofilen für Wiederansiedlungsprogramme.

## Introduction

Understanding animal movement patterns is important when designing conservation strategies (Schofield et al. 2013; Bauer et al. 2015; Buechley et al. 2018). Both space use and occurrence patterns can vary across individual life stages and contexts, including, for example, during dispersal, migration, or reproduction (Cooper and Marra 2020; Pierce et al. 2021; Heath‐Acre et al. 2022). The geographical area used by an individual, including the area in which it finds essential resources for survival and reproduction, is called its home range (Jahn et al. 2010; Abu Baker et al. 2017; Péron 2019). Home range size (HRS) is not necessarily equivalent to territory size, which is the subset of an animal’s home range that is actively defended against conspecifics (Odum and Kuenzler 1955; Anich et al. 2009; Burgos and Zuberogoitia 2020; Colombelli-Négrel et al. 2023). The factors influencing individual home range or territory size are multi-faceted, and may include age (Liu et al. 2020b), sex (Rivers et al. 2014; Alonso et al. 2020), pairing status (Mwangi et al. 2020; Sur et al. 2020) and breeding stage (Olsen et al. 2011; Liu et al. 2020a), habitat features (Hinam and Clair 2008; Arbeiter and Tegetmeyer 2011), resource availability (Karubian and Carrasco 2008; Kane et al. 2016; Rechetelo et al. 2016; Mwangi et al. 2020), and seasonal conditions (Jahn et al. 2010; van Overveld et al. 2020). Nevertheless, understanding how individual differences in behavior can influence HRS is vital for managing species’ potential for occurrence and persistence.

Consistent individual behavioral differences (hereafter “personality”) describe an individual’s response norm to social and ecological conditions, and are often associated with HRS and habitat use (Minderman et al. 2010; Stiegler et al. 2022; Stuber et al. 2022). The response norms that constitute personality must be consistent across time and contexts, and are often measured across five main axes: boldness, exploration, activity, aggressiveness, and sociability (Réale et al. 2007; Laskowski et al. 2022). Selection may favor some personality phenotypes over others (Dall et al. 2004; Réale et al. 2010), which may be associated with HRS (Stuber et al. 2022). To date, the precise mechanisms linking personality with HRS are poorly understood and may depend on the life history of the animal. For example, more exploratory individuals with a higher tolerance for novelty may travel more widely, or across a wider range of habitats, in search of food and other resources. Only a few studies have explicitly tried to link personality traits with variation in HRS. In Great Tits (*Parus major*), less aggressive males had larger home ranges (Naguib et al. 2022), suggesting that having a large home range may trade off with investment into active territory defense. By contrast, in Common Brushtail Possums (*Trichosurus vulpecula*), the correlation between exploration and HRS was positive in males but negative in females (Wat et al. 2020). Hence, although personality may influence HRS, the traits mediating this relationship may differ across species.

HRS estimates can be used to develop action plans to protect endangered species (Sawyer 2012; Morato et al. 2016; Plotz et al. 2016; Garcia-Heras et al. 2019). In many cases, target species can be elusive and difficult to track because they have low population density (Carrascal and Seoane 2009; Cosendey et al. 2016), occur over large areas (Fivaz and Gonseth 2014; Kearney et al. 2022), show migratory behavior (Hays et al. 2014; Arroyo et al. 2016), are poached (Borgerson 2015; Tarjuelo et al. 2015), or have experienced extreme habitat modification (McGowan et al. 2017; Mukul et al. 2019; Yan et al. 2021). Understanding the home range attributes of threatened species can help understand species presence and abundance and give insight into population connectivity and minimum resource requirements (Puehringer-Sturmayr et al. 2023), which is especially important in fragmented habitats. Applying principles of personality research to endangered species also has promising conservation outcomes, given the apparent association between personality and traits such as HRS and pairing success (Snekser et al. 2017; Seltmann et al. 2018). Hence, we need species-specific investigations to illuminate the relationships between personality, HRS, and parameters of biological fitness. This broader understanding of species’ needs will assist the design and implementation of conservation management plans (Haage et al. 2017). Specifically, understanding how personality phenotypes mediate reintroduction success could be a useful tool when selecting individuals for reintroduction schemes (Gotanda 2020).

The Darwin’s finches (subfamily *Geospizinae*) comprise 17 recognized species on the Galapagos Islands and are regarded as the fastest adaptive radiation of any terrestrial vertebrate (Grant and Grant 2002, 2014; Kleindorfer et al. 2022; Colombelli-Négrel et al. 2023). However, this diversity is currently threatened, with seven species categorized as vulnerable and two as critically endangered (Freile et al. 2019). Despite significant conservation interest in this unique and historically important study system, surprisingly few studies have investigated HRS in Darwin’s finches. One recent study mapped the home ranges of five VHF-tagged Medium Ground Finches (*Geospiza fortis*), a common species on Santa Cruz Island (Beausoleil et al. 2022). Across Darwin’s finch species, exploration behavior has previously been linked with diet diversity, whereby more exploratory finch species (those that showed stronger interest in a novel object) also had a more diverse diet (Tebbich et al. 2009). Therefore, we expect more exploratory finches to have larger home ranges, as they can exploit resources from a wider range of habitat across the landscape.

In this study, we investigated the association between behavioral traits and HRS in two species of Darwin’s finch: the critically endangered Medium Tree Finch (*Camarhynchus pauper*), an island-endemic species that is range-restricted to a small area (24 km^2^) of *Scalesia* forest in the highlands of Floreana Island (Dvorak et al. 2017; Peters and Kleindorfer 2018), and the Small Ground Finch (*Geospiza fuliginosa*), a common and widespread species that occurs on most islands in the archipelago (Kleindorfer et al. 2019). We used a combination of rapid-assessment cage assays (novel environment test, mirror stimulation test) and field assays (novel object test, response to broadcast of conspecific song) commonly used to measure both exploratory and aggressive behaviors in wild individuals (Réale et al. 2007; Bilby et al. 2022; Colombelli-Négrel et al. 2022; Katsis et al. 2023). We then tested for associations between the birds’ behavioral phenotype and HRS, quantified using radio telemetry. Our first prediction was that fast-exploring Darwin’s finches would have larger home ranges, aligned with what was found previously in male Common Brushtail Possums (Wat et al. 2020). Our second prediction was that more aggressive Darwin’s finches would have smaller home ranges, consistent with previous results in Great Tits and Meadow Voles (*Microtus pennsylvanicus*) (Spritzer et al. 2005; Naguib et al. 2022).

## Methods

### Study site and species

**Fig. 1:**
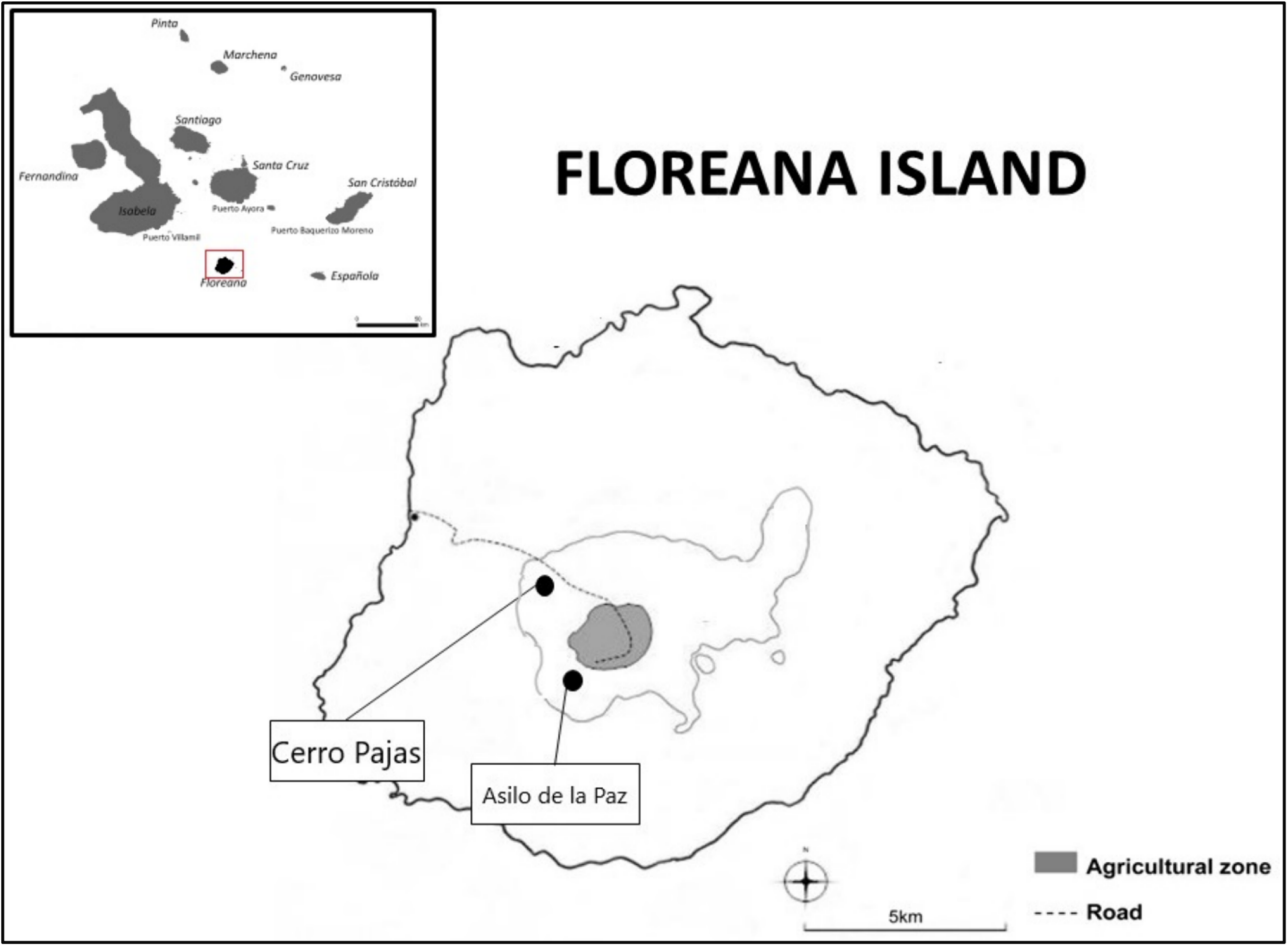
We conducted our study in the highlands of Floreana Island (dark mark in top-left inset), one of the southern islands of the Galapagos Archipelago. Our two study sites were Cerro Pajas and Asilo de la Paz (black dots)

This study was conducted on Floreana Island in the Galapagos Archipelago in 2020 (Jan–Feb) and 2022 (Feb) during the onset of the heavy rains that usually trigger nesting activity in Darwin’s finches. Both study sites, Cerro Pajas (− 1.293479, − 90.452012) and Asilo de la Paz (− 1.314003, − 90.455512), are located in the forest and woodland highlands at ~ 340 m above sea level (Fig. 1). The habitat is dominated by *Scalesia pedunculata* (family Asteraceae) and other tree species commonly used by Darwin’s tree finches for foraging and nesting (Kleindorfer et al. 2022). Rainfall levels differed substantially between the two field seasons, with mean daily rainfall of 70 mm (range 0–105 mm) during January and February 2020 and 1.5 mm (range 0–4 mm) during January and February 2022 (Galápagos Conservancy, https://www.galapagosvitalsigns.org, accessed 10. September 2022). We studied two Darwin’s finch species: the Medium Tree Finch (MTF) and Small Ground Finch (SGF). The MTF is endemic to the highlands of Floreana Island and is categorized as critically endangered by the International Union for Conservation of Nature (Freile et al. 2019), while the SGF occurs on almost all islands in the Galápagos Archipelago. Darwin’s finch populations are currently threatened by native predators, such as Short-eared Owls (*Asio flammeus galapagoensis*), introduced predators such as Feral Cats (*Felis catus*), and the introduced parasitic Avian Vampire Fly (*Philornis downsi*) (Kleindorfer et al. 2021). In our study, sample sizes for Darwin’s finch behavioral phenotype and HRS differed. This is because we conducted behavioral trials both at the time of capture and afterwards in the field (with different sample sizes for different assays, as described below), with only a subset of individuals selected for VHF tagging due to the limited number of tags available (Table S1).

### Mist-netting and radio-tagging

We used mist-nets to capture Darwin’s finches at both study sites, following protocols used to observe Darwin’s finches on Floreana Island since 2004 (Langton and Kleindorfer 2019). At the time of banding, each bird received a numbered aluminum band and a unique combination of between one and three color-bands. Shortly after capture, we measured subjects’ exploration using a novel environment test and their aggressiveness using a mirror-stimulation test (see below). We mist-netted 237 individuals from our two study species and used the first 20 MTF and first 15 SGF males for radio-tagging (in 2020: 10 MTF and 10 SGF; in 2022: 10 MTF and 5 SGF); we selected the first birds to maximize the number of field days for radiotelemetry observation. After measuring each bird’s behavioral phenotype (see below), subjects were fitted with VHF radio tags (Lotek PicoPip Ag337) that send a radio signal to a receiver (Lotek Biotracker VHF 138–168 MHz) captured by an antenna (Lotek Yagi LiteFlex). The signal had a pulse length of 12 ms, and a pulse ratio of 30 ppm. The weight of the radio tags was 0.3 g, equivalent to 1.8% of the mean MTF body mass (~ 17 g) and 2.1% of the mean SGF body mass (~ 14.4 g). The radio tags were fitted to the birds’ upper back following established protocols (Diemer et al. 2014), and subjects were released at their site of capture, typically within 1 h after mist-netting?

### Telemetry and HRS

We located each tagged Darwin’s finch during a period of three to seven non-consecutive days (mean ± SE = 4.0 ± 0.2 days) until we obtained at least 20 fixes per bird, aiming that each GPS point was separated by a minimum of 30 min (some of them shorter) (Streby et al. 2015). We reached this threshold for 26 individuals (74% of tracked males) within four non-consecutive days of tracking. One additional bird fell short of this threshold (13 fixes) due to the loss of his transmitter. We retained this individual in our analysis, given that our results did not change when he was excluded. Radio telemetry tracking (hereafter “tracking”) was conducted in the morning between 06:00 and 12:00 GALT. In total, we obtained 822 fixes from 35 males (20 during the 2020 season, 15 during the 2022 season; Fig. S1), with a mean ± SE of 23.5 ± 0.5 fixes per bird (range 13–27) and 5.5 ± 0.3 fixes per individual per day (range 1–12). We collected 464 fixes from 20 MTF (10 from 2020, 10 from 2022), with 23.2 ± 0.7 fixes (range 13–26) per individual and 358 fixes from 15 SGF (10 from 2020, 5 from 2022) with a mean of 23.9 ± 0.5 SE fixes per individual (range 13–27). The mean ± SE interval between fixes collected within the same day was 43.8 ± 0.9 min (range 22–314 min). We use the *hrBootstrap* function from the package *adehabitatHR* to estimate the number of fixes needed, where we observed how the trend starts to flattened after 13 to 14 fixes, which suggests that enough fixes were taken for the individuals (Fig. S7).

### Novel environment test (exploration)

Exploration was measured during short-term captivity using a novel environment test (*n* = 44 MTF, 59 SGF; see Table S2 for summary based on species, pairing status, and year). After capture and processing, subjects were placed individually into a plastic release box (19 × 14 × 10 cm) for a standardized acclimation period (Bilby et al. 2022; Katsis et al. 2024). All finches tested in a given year experienced the same acclimation period: 5 min in 2020 and 1 min in 2022 (shortened to reduce waiting times for captured birds). This difference in acclimation period did not influence individuals’ behavioral response, either in the novel environment test (*t* test for unique sector visits: *n* = 101, *t* = 1.7, *df* = 65.2, *p* = 0.097) nor the mirror stimulation test (*t* test for *mirror attacks; n* = 101, *t* = -0.9, *df* = 35.1, *p* = 0.387). After the door to the release box was raised, the bird was allowed to enter the novel environment: a metal flight cage (75 × 44 × 42 cm) with three perches (one at height 6 cm and the other two at height 20 cm) (Fig. S2). The novel environment contained 13 discrete sectors that the subject could visit (three perches, four floor quadrats, four cage walls, the cage ceiling, and the release box). The novel environment test lasted 5 min, during which we recorded*:* (a) the number of unique sector visits, and (b) the number of total sector visits (including repeat visits). Although the repeatability of exploration has not been tested in Darwin’s finches, the trait is known to be individually consistent across several songbird species, including Great Tits (*Parus major*; Thys et al. 2017), Australian Zebra Finches (*Taeniopygia castanotis*; McCowan et al. 2015), Common Starlings (*Sturnus vulgaris*; Thys et al. 2017), and Superb Fairywrens (*Malurus cyaneus*; Hall et al. 2015). The novel environment test in our study was identical to that previously used by Katsis et al. (2023), who found that exploration was significantly repeatable in adult Superb Fairywrens. In a recent study, we demonstrated that exploration behavior differs between Darwin’s finch species and predicts such factors as territory defense and offspring hatching success, supporting the ecological relevance of this trait (Katsis et al. 2024).

### Mirror simulation test (aggressiveness)

We measured aggressiveness during short-term captivity using a mirror stimulation test. Immediately after the novel environment test, we remotely raised a curtain to reveal a mirror at one end of the flight cage (Bilby et al. 2022; Katsis et al. 2024) (Fig. S2). We expected male Darwin’s finches to respond aggressively to their mirror image, as they are highly territorial during the breeding season and will defend against conspecific intruders (Ratcliffe and Grant 1983). Over a 3-min period, we then recorded (a) time near mirror, the duration (in seconds) spent within the three nearest sectors to the mirror, excluding time spent attacking the mirror; and (b) number of mirror attacks, the number of times the subjects made physical contact with the mirror. In songbirds, proximity to and physical contact with the mirror is generally interpreted as an aggressive response (Leitão et al. 2019; Jones et al. 2022) and this response has been shown to be significantly repeatable in superb fairy-wrens (Katsis et al. 2023). The novel environment and mirror stimulation tests were recorded with a GoPro camera placed 50 cm outside the flight cage and behavior was scored using the software Solomon Coder v. beta 19.08.02 (András Peter, https://solomon.andraspeter.com).

### Novel object test (exploration)

We measured exploration in the field as the neophilic response to a novel object placed in their habitat, adapting protocols previously used by Tebbich et al. (2009). First, we placed one of three different novel objects (30 × 50 cm double metal grid, 20 × 50 cm yellow bag, or 20 × 50 cm orange bag) in the field at height 2 m in natural vegetation. We placed small stones and branches to demarcate a 10-m radius around the novel object. Birds were alerted to the start of the trial using a bird whistle (Audubon, model number MT-PN-28905150) blown from a position 15 m from the novel object. The trial lasted 15 min and we scored birds’ response to the novel object as the latency (in min) to approach within 10 m of the novel object. To aid interpretation of this variable, we then inverted the latency score by subtracting each latency from the maximum possible value (15 min), so that higher scores indicated a higher level of exploration: that is, birds approaching in min 1 received a score of 15–1 = 14 (high exploration) and birds approaching in minute 14 received a score of 15–14 = 1 (low exploration). Of the 35 MTF and 100 SGF that approached the novel object in the wild during the field season in 2022 (García-Loor et al., unpublished data), 15 were color-banded and had previously been assayed using the novel environment test (8 MTF, 7 SGF) either in 2020 or 2022, allowing us to validate our measures of exploration across contexts.

### Simulated conspecific intrusions (aggressiveness)

We measured aggressiveness in the field using simulated conspecific intrusions (song playback) in males’ territories (15 MTF, 20 SGF). We targeted color-banded birds whose behavior we had previously measured in the novel environment and mirror stimulation tests. We prepared song playback tracks using conspecific songs recorded in 2020 with a good signal-to-noise ratio (Colombelli-Négrel et al. 2023). We used nine unique playback tracks for the 15 MTF trials and 18 unique playback tracks for the 20 SGF trials. Each track had a duration of 3 min, comprising 1 min of playback followed by 1 min of silence and 1 min of playback. Each minute of playback contained six songs spaced at 10-s intervals. We conducted all playback trials between 06:00 and 11:00 GALT, corresponding with the peak of song activity. After observing the male singing within 10 m from his nest, we placed the speaker (Sony XB12 Extra Bass Portable Bluetooth Speaker, Sony Australia Limited or Soundcore Icon Mini Bluetooth Speaker, Anker Technology Ltd., United Kingdom; frequency response 20 Hz–20 kHz) and iPod on the ground 5 m from the nest and broadcast a randomly chosen playback track of a conspecific song (played at ~ 80 dB at 1 m; measured with a VLIKE VL6708 sound-level meter). One observer located ~ 10 m from the speaker narrated the trial into a directional microphone connected to a digital audio recorder. During the 3-min playback trial, we recorded the following behaviors: (a) time within 1 m, the duration (in seconds) spent within 1 m of the speaker; (b) minimum distance, the minimum distance (in m) between the focal bird and the speaker; (c) flights, the number of flights within 20 m of the speaker, and (d) crosses, the number of flights over the speaker (Colombelli-Négrel et al. 2023).

### Nest monitoring and pairing status

Although we started tracking only unpaired males, some subjects paired up during the observation period; therefore, we controlled for pairing status in our analysis and expected to find the same patterns of association between HRS and behavioral traits irrespective of pairing status. Nests were monitored following standardized protocols that were developed in 2004 on Floreana Island and maintained in the years since (Kleindorfer 2007; O'Connor et al. 2010; Kleindorfer et al. 2021). The study areas were divided into twelve study plots (each 200 × 100 m) that were systematically searched for singing males, nest building, and nest occupation. In addition, the pairing status of VHF-tagged males was confirmed by repeated observations at the nest during radiotracking, with males subsequently classed as paired (female seen lining the nest or incubating eggs) or unpaired (male sang without an observed female) (Kleindorfer et al. 2021). In total, we measured HRS for 35 males that were unpaired at the time of banding, of which 23 remained unpaired (15 MTF, 8 SGF) and twelve became paired (5 MTF, 7 SGF) within 7 days (i.e., the duration of the radio tracking). In Darwin’s finches, males build a display nest and sing at the nest to attract a female, but about 50% of singing males at a display nest can remain unpaired when monitored across 1 month (Kleindorfer et al. 2021).

### Statistical analysis

We performed statistical analyses using the software R version 4.1.1 (R Core Team 2022). To calculate male HRS, we used the package *ctmm* version 1.2.0 to obtain the kernel density estimates at 95% range, considering the model type of the independent identically distributed (IID) process (Fieberg 2007; Calabrese et al. 2016; Signer et al. 2019). Principal component analysis (PCA) was executed using the function *princomp* (R package *stats* version 3.6.2). All the Spearman’s correlation tests (as variables were not normally distributed) were executed using the function *cor.test* (*stats* version 3.6.2). Linear models and generalized binary logistic regression were performed using the function *lm* and *glm* (*stats* version 3.6.2). We based our model selection on AIC values, keeping the model with the lower value, when ΔAIC was > 2 points (Burnham and Anderson 2002); therefore, we include interactions in some models but not others. We checked for influential observations by calculating Cook’s distance using the function *cooks.distance* (*stats* version 3.6.2); we didn’t find any highly influential observations, with all Cook’s distance scores < 0.2. We determined variance inflation factor (VIF) using the function *vif* from the R package *car* (version 3.0–13; Fox and Weisberg 2011), confirming that there were no collinearity issues in the model (highest VIF = 1.19).

#### (a) Creation of PC_Aggressiveness variable

During the simulated territory intrusions, we measured four male response variables: time within 1 m, minimum distance, flights, and crosses. We used PCA to reduce these four variables to one uncorrelated principal component (PC_Aggressiveness) with an eigenvalue of 3.08 and explaining 77% of total variance. Higher PC_Aggressiveness values indicated a more aggressive response to conspecific playback. To normalize the residuals in our linear model, all four variables were square-root-transformed prior to running the PCA. Factor loadings for the four response variables were: time at 1 m (0.47), minimum distance (− 0.52), flights (0.52), and crosses (0.49).

#### (b) Validation of behavioral traits

For each behavior (exploration and aggressiveness), we compared two behavioral responses, both within and between assays, using Spearman’s correlation tests (as variables were not normally distributed). For exploration, we tested the consistency of behavioral variables (1) within the novel environment test, comparing the number of unique sector visits vs total sector visits) and (2) across contexts, by comparing exploration in the novel environment (unique sector visits) with exploration towards a novel object in the field (latency to approach to 10 m). For aggressiveness, we tested the consistency of behavioral variables (1) within the mirror simulation test, by comparing time near mirror between birds that did and did not contact the mirror (paired *t* test and generalized binary logistic regression) and (2) across contexts, by comparing aggressiveness score from the mirror stimulation test (time near mirror) against the derived aggression scores obtained during the simulated conspecific intrusions (PC_Aggressiveness).

#### (c) Behavioral traits and HRS

We estimated the association between behavioral traits and HRS in both finch species, using both exploration (*n* = 34 individuals; 19 MTF, 15 SGF) and aggressiveness (*n* = 33 individuals; 19 MTF, 14 SGF). We log-transformed HRS values prior to analysis to normalize the model residuals. Since we predicted that more exploratory and less aggressive individuals would occupy larger home ranges, we included both behavioral scores and species in the main model [*lm(log10(HRS)* ~ *Exploration (unique sector visits)* + *Aggressiveness (time near mirror)* + *Species*]. In an alternative model, we also controlled for the males’ pairing status [*lm(log10(HRS)* ~ *Exploration (unique sector visits)* + *Aggressiveness (time near mirror)* + *Species* + *Status*].

## Results

Mean ± SE HRS (level 95%) was 1.8 ± 0.4 ha (range 0.2–7.0) in MTF and 2.3 ± 0.6 ha (range 0.1–8.9) in SGF, with no difference between species (*n* = 35, *t* = − 0.7, *df* = 23.4, *p* = 0.52). When considering both species together, paired males occupied smaller home ranges than unpaired males (Table 1; paired *t* test; *n* = 35, *t* = − 3.1, *df* = 26.3, *p* = 0.004). Analyzing each species separately, paired and unpaired MTF males did not differ in the size of their home ranges (*n* = 20, *t* = − 1.5, *df* = 17.8, *p* = 0.10), while unpaired SGF males had five times larger home ranges than paired males (*n* = 15, *t* = − 2.7, *df* = 7.6, *p* = 0.03).

**Table 1:** Summary of home range sizes (ha) in male Medium Tree Finches (MTF) and Small Ground Finches (SGF)

	All			2020			2022		
	*n*	Mean ± SE	Range	*n*	Mean ± SE	Range	*n*	Mean ± SE	Range
MTF				10	0.9 ± 1.9	0.2–1.9	10*	2.7 ± 0.7	0.4–7.0
Unpaired	15	2 ± 0.5	0.2–7	5	0.7 ± 0.3	0.2–1.9			
Paired	5	1.1 ± 0.3	0.2–1.7	5	1.1 ± 0.3	0.2–1.7			
	All			2020			2022		
	*n*	Mean ± SE	Range	*n*	Mean ± SE	Range	*n*	Mean ± SE	Range
SGF				10	1.4 ± 0.6	0.1–6.8	5*	4.1 ± 1.4	0.9–8.9
Unpaired	8	3.7 ± 1.1	0.8–9	3	3 ± 1.9	0.8–6.8			
Paired	7	0.7 ± 0.2	0.1–1.8	7	0.7 ± 0.6	0.1–1.8			

The table shows sample size (*n*), mean ± SE, and range (min to max) of home range sizes, grouped by species (MTF, SGF), pairing status (paired, unpaired) and year (2020, 2022). *In 2022, all monitored males built a nest, but none found a breeding partner during the 4-week study period

### (a) Validation of behavioral traits

**Fig. 2:**
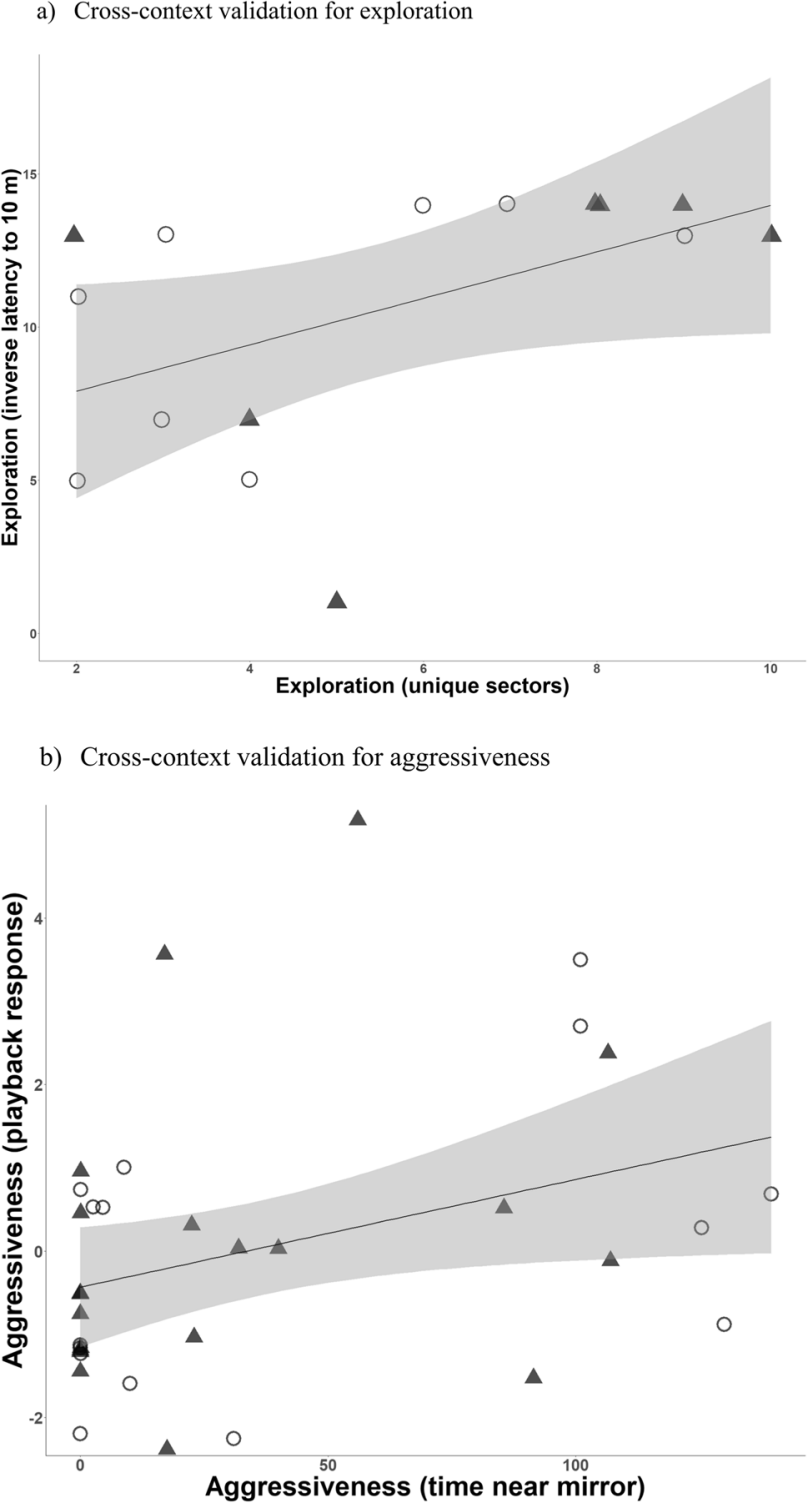
Behavioral variables measured in the same individuals across contexts in Medium Tree Finches (open circles) and Small Ground Finches (filled triangles). **a** Exploration in the novel environment test (number of unique sectors visites) correlated positively with exploration in the novel object test (inverse latency to approach to 10 m), when considering both species together (*r* = 0.6, *n* = 15, *p* = 0.03). **b** Aggressiveness during the mirror simulation test (time near mirror) correlated positively with aggressiveness during a simulated territory intrusion (playback response, PC_Aggressiveness) when considering both species together (*r* = 0.4, *n* = 35, *p* = 0.04)

Exploration variables within the novel environment test (unique sector visits and total sector visits) were strongly positively correlated in MTF (*n* = 44, *r* = 0.8, *p* < 0.001), in SGF (*n* = 59, *r* = 0.7, *p* < 0.001), and overall in both species (*n* = 103, *r* = 0.8, *p* < 0.001) (Fig. S3). Therefore, in future analyses we used unique sector visits as a proxy for exploration in the novel environment test. Across contexts, exploration of the novel environment (unique sector visits) and towards the novel object (latency to approach to 10 m) were significantly positively correlated when pooling data for both species (*n* = 15, *r* = 0.6, *p* = 0.032), but not when considering MTF (*n* = 8, *r* = 0.6, *p* = 0.106) or SGF (*n* = 7, *r* = 0.5, *p* = 0.263) separately (Fig. 2a).

For the aggressiveness variables measured in the mirror stimulation test, males from both species that attacked the mirror also spent more time near the mirror (*n* = 10, mean: 84.5 ± 15.1 SE; range 3–139.5) compared to males that did not attack the mirror (*n* = 55, mean: 44.8 ± 5.09 SE; range 0.4–132.5) (*n* = 65, *t* = 2.6, *df* = 11.1, *p* = 0.026). This pattern was also significant in the binary logistic regression model (*z* = 3.707, *p* < 0.001) (Fig. S4). Across contexts, aggressiveness towards the mirror (time near mirror) and towards conspecific playback (PC_Aggressiveness) were significantly positively correlated when pooling both species (*n* = 35, *r* = 0.4, *p* = 0.031), but not when considering MTF (*n* = 15, *r* = 0.5, *p* = 0.091) or SGF (*n* = 20, *r* = 0.3, *p* = 0.212) separately (Fig. 2b).

### (b) Behavioral traits and HRS

**Fig. 3:**
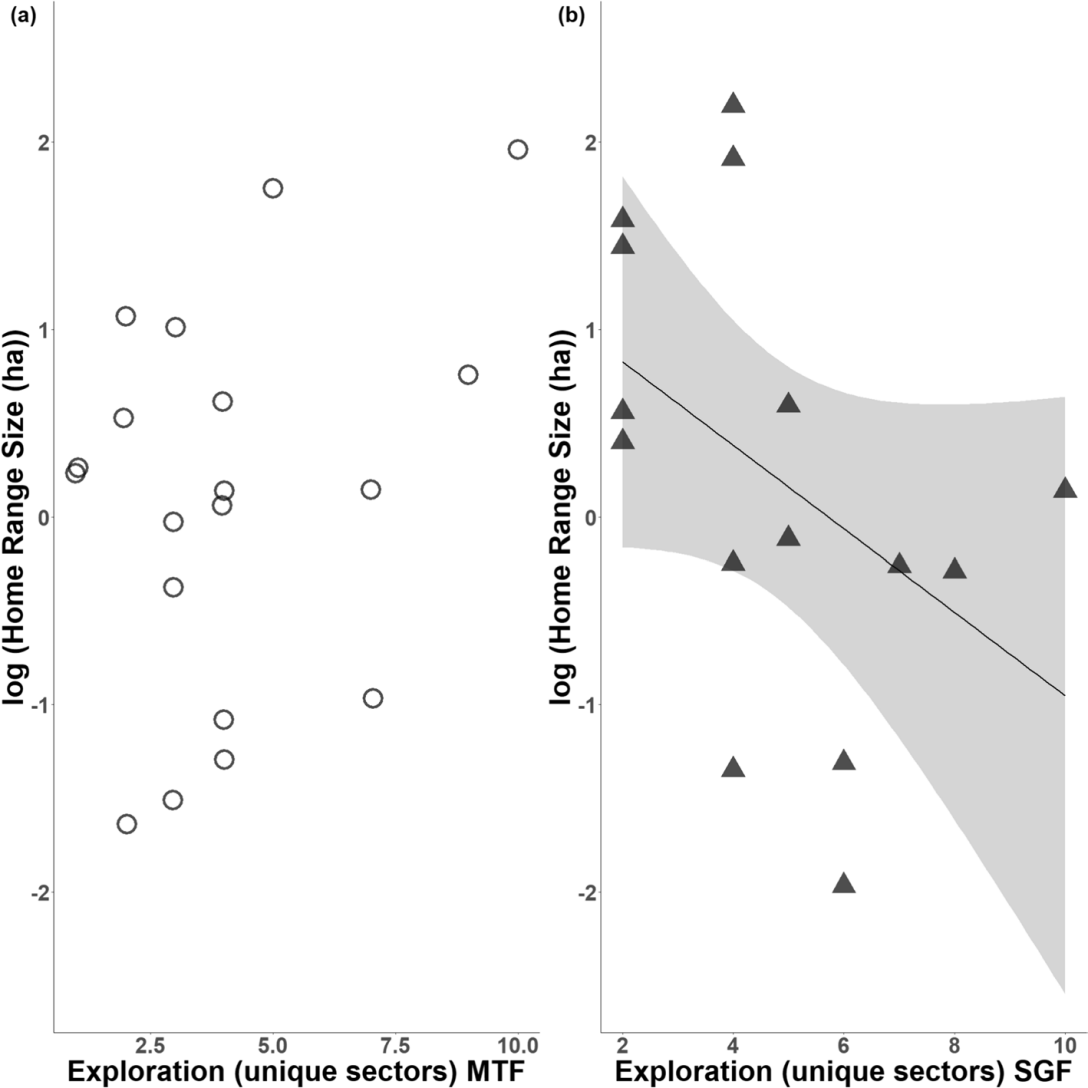
Relationship between exploration and home range size (log-transformed) differed between Darwin’s finch species. The number of unique sectors visits during a novel environment test **a** did not predict home range size in Medium Tree Finches, and **b** was negatively associated with home range size in Small Ground Finches. 95% confidence intervals are shown in grey

In MTF, there was no significant association between HRS and exploration (unique sector visits, *n* = 19, *r* = 0.2, *p* = 0.486; total sector visits, *n* = 19, *r* = − 0.1, *p* = 0.613). However, in SGF males, more exploratory birds in the novel environment had significantly smaller home ranges when considering unique sector visits as a measure of exploration (*n* = 15, *r* = − 0.6, *p* = 0.033) (Fig. 3); but not when considering total sectors visits (*n* = 15, *r* = -0.43, *p* = 0.109).

**Fig. 4:**
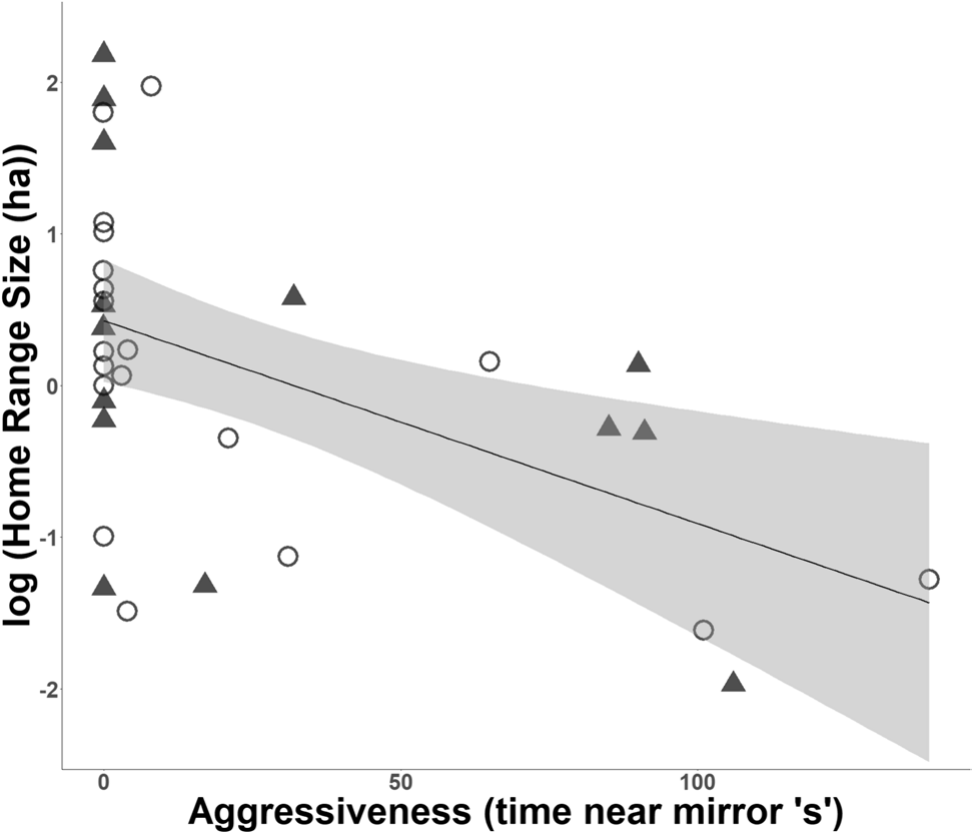
Aggressiveness in the mirror stimulation test (time near mirror, in secs) was negatively associated with home range size (log-transformed) in Medium Tree Finches (open circles) and Small Ground Finches (filled triangles) (*r* = − 0.5, *n* = 33, *p* = 0.001). 95% confidence intervals are shown in grey

Aggressiveness (time near mirror) was negatively associated with HRS in both species overall (*n* = 33, *r* = − 0.5, *p* = 0.001), as well as in MTF (*n* = 19, *r* = − 0.5, *p* = 0.017) and SGF (*n* = 14, *r* = − 0.5, *p* = 0.050) separately. That is, males that spent longer near the mirror had smaller home ranges (Fig. 4). Our linear model showed that aggressiveness (time near mirror) was a significant predictor of HRS (*t* = − 3.340, *p* < 0.01*, r*^*2*^_*adj*_ = 0.20) over exploration (unique sector visits) or species (Table 2). In a separate set of models, pairing status did not significantly predict HRS and the negative relationship between aggressiveness (time near mirror) and HRS remained significant when pairing status was included in the model (*t* = − 2.858, *p* < 0.01*, r*^*2*^_*adj*_ = 0.24) (Table S3). We also found a near-significant negative trend between aggressiveness in the wild (simulated conspecific intrusions) and HRS when analyzing both species together (*n* = 20, *r* = − 0.4, *p* = 0.098) (Fig. S6).

**Table 2:** Output from linear models testing the effects of personality and species (Medium Tree Finch, MTF, or Small Ground Finch, SGF) on home range size (log-transformed)

	Estimate	Std. Error	*t*	*p*
(Intercept)	0.056	0.371	0.151	0.881
Unique sector visits	0.080	0.076	1.056	0.300
Time near mirror	− 0.015	0.005	− 3.340	**0.002**
Species (SGF)	0.138	0.351	0.392	0.698

“Unique sector visits” is a measure of exploration during the novel environment test (with higher values indicating greater exploration) and “time near mirror” is a measure of aggressiveness during the mirror stimulation test (with higher values indicating greater aggressiveness)

## Discussion

In SGF and MTF on Floreana Island, our measures of aggressiveness and exploration were consistent both within and across assay types, supporting the consistency of between-individual behavioral differences. Those differences, in turn, predicted individuals’ HRS. Firstly, in both species, more aggressive males occupied smaller home ranges. Second, the association between exploration behavior and HRS differed between species: in MTF, exploration did not predict HRS, while in SGF more exploratory males had smaller home ranges. These different patterns may be related to differences in selection pressures for both species, since MTF are a habitat-restricted specialist while SGF are a widespread generalist that occurs in most habitat types on the Galapagos Archipelago (Kleindorfer et al. 2022).

### Home range size

To date, only one published study has used radiotelemetry to assess HRS in a Galapagos landbird. Beausoleil et al. (2022) used telemetry to estimate the home range of five Medium Ground Finch individuals (two males, three females) during the breeding season in a single year (2019). This study was undertaken in the lowlands of Santa Cruz Island at *El Garrapatero* beach (dry habitat) and reported home ranges that were about 10 times larger than those for SGF in the Floreana highlands. Clearly, many factors differed across the two studies, including study species, year, island, and habitat type. On Floreana Island, SGF home ranges are four times larger in the dry lowlands than in the humid highlands (García-Loor et al., unpublished data), suggesting that habitat and resource availability helps to shape individuals’ movement patterns. In their Medium Ground Finch study, Beausoleil et al. (2022) did not consider individual behavioral traits, which could perhaps have explained additional variation in subjects’ HRS. The marked difference in HRS across studies underscores the need for island-specific information, especially for range-restricted species of conservation concern.

### Exploratory behavior and HRS

Contrary to our predictions, more exploratory SGF males occupied smaller rather than larger home ranges. This finding conflicts with a previous study in Common Brushtail Possums, in which more exploratory males had larger home ranges, although this association between exploration and HRS was sex-dependent (Wat et al. 2020). Future work should seek to understand the mechanisms that underlie these species-specific associations between behavioral traits and HRS. For example, one could measure the ontogeny of personality across life stages (Katsis et al. 2023) and how behavioral changes across an individual’s lifespan influence its dispersal and spatial use patterns. Darwin’s finches are socially monogamous and provide biparental care to offspring both during the nestling stage (Kleindorfer et al. 2022) and for about 2-week post-fledgling, after which time juvenile finches tend to flock and roost together (Schluter 1982; Beausoleil et al. 2022). During this formative period, juvenile social aggregations may affect individuals’ foraging behavior and, hence, their long-term spatial behavior. Indeed, in captive Australian Zebra Finches, younger individuals develop similar foraging behavior to adults in close proximity through social learning (Farine et al. 2015). In addition to social factors, ecological factors such as predator abundance and food availability may also shape personality distributions at the population level, with shifting selection pressures across islands and seasons (Boag and Grant 1984).

### Aggressive behavior and HRS

In both species, we found a negative association between male aggressiveness and HRS. Our results are aligned with previous findings in Great Tits and in Meadow Voles, whereby individuals that were more aggressive, and hence less tolerant of intruders, occupied smaller home ranges (Spritzer et al. 2005; Naguib et al. 2022). If more aggressive males are better at outcompeting conspecifics, this may allow them to occupy high-quality patches that contain all required resources within a relatively small area. Conversely, less aggressive males may be forced to travel more widely to obtain the resources that they need. Future research should aim to identify the habitat characteristics that mediate differences in HRS. In addition, it would be informative to test whether females prefer to pair with aggressive males and, if so, whether this preference generates direct or indirect fitness benefits. From a physiological perspective, aggressiveness is linked to high testosterone levels (Wingfield et al. 1990; Smith et al. 2005). In socially monogamous bird species, both testosterone levels and agonistic behavior tend to decrease during offspring rearing phase, as observed in Lapland Longspurs (*Calcarius lapponicus*) (Hunt et al. 1997). In Darwin’s finches, males and females do not differ in their levels of the stress hormone corticosterone levels during the breeding season (Clark et al. 2018). Future work should examine the relationship between aggressiveness and hormone production across breeding stages in Darwin’s finches, as previous studies have shown that opportunistic breeders, such as Darwin’s finches, can regulate the starting of their annual reproductive events based on weather conditions (Hau et al. 2004).

In both Darwin’s finch species, paired males occupied smaller home ranges than unpaired males, which suggests that males may reduce their movements post-pairing to more vigorously defend their territory and/or guard their mating partner. However, in our study, it is difficult to disentangle the causality of this relationship: do male finches reduce their home ranges upon pairing, or are males with small home ranges more likely to secure a mate? Females may prefer males with smaller home ranges if, for example, these males are spending more time displaying at their nest, or if their smaller home ranges contain a greater proportion of high-quality habitat (e.g., Favaron et al. 2006).

### Conservation implications

Understanding the movement patterns of threatened species can provide relevant information for conservation management (Puehringer-Sturmayr et al. 2023). From a foraging and habitat use perspective, generalist species occupy larger home ranges and tend to be more resilient under conditions of habitat loss, whereas specialist species are more vulnerable to habitat loss and generally experience a higher risk of extinction (Owens and Bennett 2000; Slatyer et al. 2013; Castañeda et al. 2019; Guerrero‐Sanchez et al. 2022). The generalist SGF is a common and widespread species whose distribution has expanded in recent decades from the lowlands to the highlands on both Santa Cruz (Galligan and Kleindorfer 2010; Sulloway and Kleindorfer 2013) and Floreana Island (O’Connor et al. 2010; Kleindorfer et al. 2021). Its higher exploration scores in our study are consistent with the predictions of the neophobia threshold hypothesis, which proposes that more generalist species should be less wary of novelty (Greenberg 1983, 1990). The MTF has a more specialist diet and is a range-restricted species endemic to the Floreana highlands, categorized as critically endangered (Freile et al. 2019; Kleindorfer et al. 2022). In the latter species, understanding movement patterns is essential for developing conservation strategies and management plans, especially given the limited habitat available to this species, representing only 26% (4.599 ha) of the island, of which 5% (230 ha) is used for agriculture (Island Conservation and Galapagos National Park Directorate 2020). In our study, Medium Tree Finches were tagged only in the *Scalesia* forest that covers the island’s central highland and generally remained in this area. However, the species is also currently establishing itself in the adjacent agricultural areas, outside of the protected land of Galapagos National Park, and future studies should aim to map the home ranges and movement patterns of these individuals in the agricultural zone.

### Limitations of the study

One limitation of our study is that we do not have repeated testing of behavioral variables over time, which prevents us from calculating the temporal consistency of exploration and aggressiveness (Niemelä and Dingemanse 2018). Without data on within-individual behavioral variation, we cannot distinguish between phenotypic correlations that occur within and between individuals, which risks under- or over-estimating the correlation between behavior and HRS (Niemelä and Dingemanse 2018). Nevertheless, we provide some evidence that between-individual behavioral differences are consistent across contexts (i.e., across two different assays measuring the same purported personality trait) for both exploration and aggressiveness, partially satisfying the definition of personality as “consistent between-individual differences in behaviour across time and/or contexts” (Sánchez-Tójar et al. 2022).

## Conclusion

This study found within- and between-context support for consistent behavioral differences in exploration and aggressiveness behavior in two Darwin’s finch species studied across 2 years. Our measurements of behavioral traits, which align with two major personality axes (exploration and aggressiveness), predicted individuals’ HRS. In both species, males that were more aggressive (spent more time near the mirror during a mirror stimulation test) had smaller home ranges. The relationship between exploration and HRS differed between species, which may be related to differences in foraging ecology between the SGF (a fast-exploring generalist) and MTF (a slower-exploring invertebrate specialist). From a conservation perspective, using our understanding of HRS to inform conservation management will be of increasing importance as we attempt to redress the impacts of the Anthropocene (Lewis and Maslin 2015) and biodiversity crisis (Koh et al. 2004; Rodríguez-Rodríguez and Martínez-Vega 2022). On Floreana Island, the Galapagos National Park Directorate has begun implementing a large-scale eradication program of introduced mammals to reverse the island’s significant biodiversity losses (Hanson and Campbell 2020). Therefore, having baseline data on the Darwin’s finches’ behavioral and spatial ecology prior to the eradication creates opportunity to compare changes in personality structure, breeding success, and movement patterns after removal of invasive predators (Banko et al. 2019).

## Supplementary Information

Below is the link to the electronic supplementary material.

Supplementary file1 (DOCX 4508 KB)

## Data Availability

The movement data for this manuscript are available on Movebank (https://www.movebank.org), you can look to it referring to the principal investigator: Sonia Kleindorfer—Konrad Lorenz Research Center, the data us is open to public access. The personality data and the RStudio script are available on the repository PHAIDRA from the University of Vienna (https://phaidra.univie.ac.at/).
