# Supplementary material for: Aggressive behavior as a predictor of home range size: findings from both range-restricted and widespread Darwin’s finch species
Source: J Ornithol. 2024 Sep 21;166(1):247–61. doi: 10.1007/s10336-024-02215-7 (PMC11779779; doi:10.1007/s10336-024-02215-7)

**Supplementary material**

**Aggressive behavior as a predictor of home range size: findings from both range-restricted and widespread Darwin’s finch species**

Jefferson García-Loor^1,2^, Mario Gallego-Abenza^2,3^, Andrew C. Katsis^1,2,4^, Verena Puehringer-Sturmayr ^2^, Diane Colombelli-Négrel^4^, Çağlar Akçay^5,6^, Sonia Kleindorfer^1,2,4^

^1^ Department of Behavioral and Cognitive Biology, University of Vienna, 1030, Austria

^2^ Konrad Lorenz Research Center for Behavior and Cognition, University of Vienna, 4645, Austria

^3^ Department of Zoology, Stockholm University, Stockholm, Sweden

^4^ College of Science and Engineering, Flinders University, Adelaide, 5001, Australia

^5^ School of Life Sciences, Anglia Ruskin University, Cambridge, United Kingdom

^6^ Department of Psychology, Koç University, Istanbul, Türkie

# Corresponding author:

Jefferson García-Loor, [garcialoorjefferson@gmail.com](mailto:garcialoorjefferson@gmail.com)

**Table S1** Sample sizes for each behavioral assay (data type) in the study are shown for both focal species as medium tree finch / small ground finch. The diagonals indicate the total number of individuals for each of the assays whereas the cells above indicate sample sizes of individuals tested for both of the respective assays.

|  | Radiotracking | Exploration Cage | Exploration Wild | Aggressiveness Cage | Aggressiveness Wild |
| --- | --- | --- | --- | --- | --- |
| Radiotracking | 20/15 | 19/15 | 5/3 | 19/14 | 8/12 |
| Exploration Cage |  | 44/59 | 8/7 | 44/59 | 15/20 |
| Exploration Wild |  |  | 8/7 | 8/7 | 2/2 |
| Aggressiveness Cage |  |  |  | 42/59 | 15/20 |
| Aggressiveness Wild |  |  |  |  | 15/23 |

**Table S2** Summary of Darwin’s finch personality variables per species (medium tree finch = MTF, small ground finch = SGF) in paired and unpaired males per year. Data are shown with sample size (n), mean, standard error (SE) and range classified by species, year and pairing status. *Unique sector visits* and *total sector visits* are measures of exploration (novel environment test), while *time near mirror* is a measure of aggressiveness (mirror stimulation test). All monitored finches remained unpaired during the 2022 field season.

|  |  | **2020 Paired** | | | **2020 Unpaired** | | | **2022 Unpaired** | | |
| --- | --- | --- | --- | --- | --- | --- | --- | --- | --- | --- |
| **MTF** |  | **n** | **Mean ± SE** | **Range** | **n** | **Mean ± SE** | **Range** | **n** | **Mean ± SE** | **Range** |
|  | Unique Sector Visits | 5 | 2.8 ± 1.11 | (1–7) | 5 | 3.6 ± 0.4 | (2–4) | 9 | 5.1 ± 0.96 | (2–10) |
|  | Total Sector Visits | 5 | 23.6 ± 14.2 | (1–70) | 5 | 21.4 ± 11.9 | (2–65) | 9 | 33.9 ± 15.5 | (2–113) |
|  | Time Near Mirror | 5 | 14.8 ± 12.7 | (0–65.5) | 5 | 54.9 ± 27.9 | (0–139.5) | 9 | 3.4 ± 2.4 | (0–21.2) |
| **SGF** |  | **n** | **Mean ± SE** | **Range** | **n** | **Mean ± SE** | **Range** | **n** | **Mean ± SE** | **Range** |
|  | Unique Sector Visits | 7 | 6.6 ± 0.65 | (4–10) | 3 | 3.3 ± 0.67 | (2–4) | 5 | 3 ± 0.6 | (2–5) |
|  | Total Sector Visits | 7 | 70.3 ± 16.5 | (16–50) | 3 | 9.7 ± 6.23 | (2–22) | 5 | 3.4 ± 0.9 | (2–6) |
|  | Time Near Mirror | 7 | 60.4 ± 16.1 | (0–106.5) | 3 | 0 | (0) | 4 | 0 | (0) |

**Table S3** Output from linear models testing whether exploration (unique sector visits) or aggressiveness (time near mirror) predicts home range size in medium tree finches (MTF) and small ground finches (SGF). Species (MTF, SGF) and pairing status (paired, unpaired) were included as fixed effects.

|  | **Estimate** | **Std. Error** | **t** | **p** |
| --- | --- | --- | --- | --- |
| (Intercept) | -0.425 | 0.481 | -0.884 | 0.384 |
| **Unique Sector** | 0.085 | 0.074 | 1.147 | 0.261 |
| **Time Near Mirror** | -0.013 | 0.005 | -2.858 | **0.008** |
| Species (SGF) | 0.249 | 0.351 | 0.710 | 0.484 |
| Status (Unpaired) | 0.572 | 0.375 | 1.526 | 0.138 |

**Fig. S1** Sightings, or so-called ‘tracking fixes’, of medium tree finches (MTF) and small ground finches (SGF) on Floreana Island. To project and plot the fixes, we used the packages ‘moveVis’ version 0.10.9 (Schwalb-Willmann et al. 2020), ‘move’ version 4.2.4 (Kranstauber et al. 2023), and ‘RgoogleMaps’ version 1.4.5.3 (Loecher and Ropkins 2015). a) Tracking fixes plotted on a map of Floreana Island, with each color representing a tracked individual. *N* = 822 fixes from 35 males (21 medium tree finches, 14 small ground finches). b) The polygons of the occupancy area of the tracked individuals at both study sites.

1. Image of tracking ‘fixes’


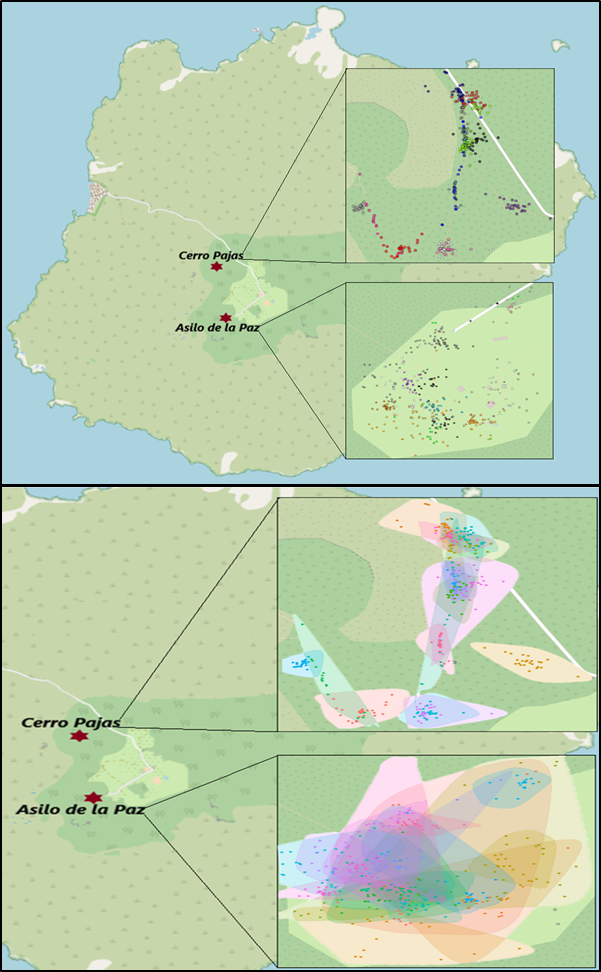


1. Image of home range polygons


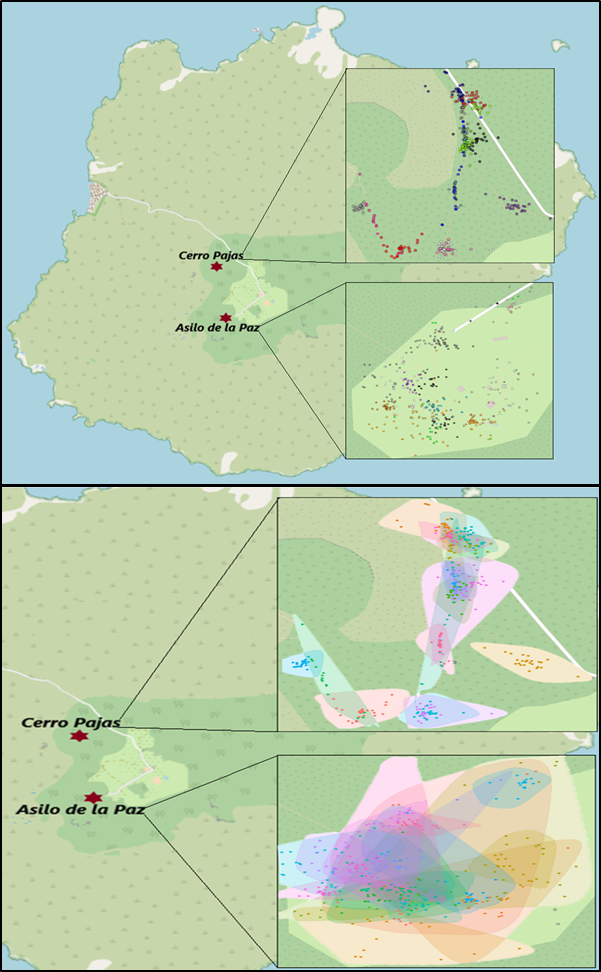


**Fig. S2** Picture of the metal flight cage (75 × 44 × 42 cm) with three perches (one at 6 cm height and the other two at 20 cm height) where the novel environment test and the mirror simulation test was performed. Photo take by Andrew Katsis.


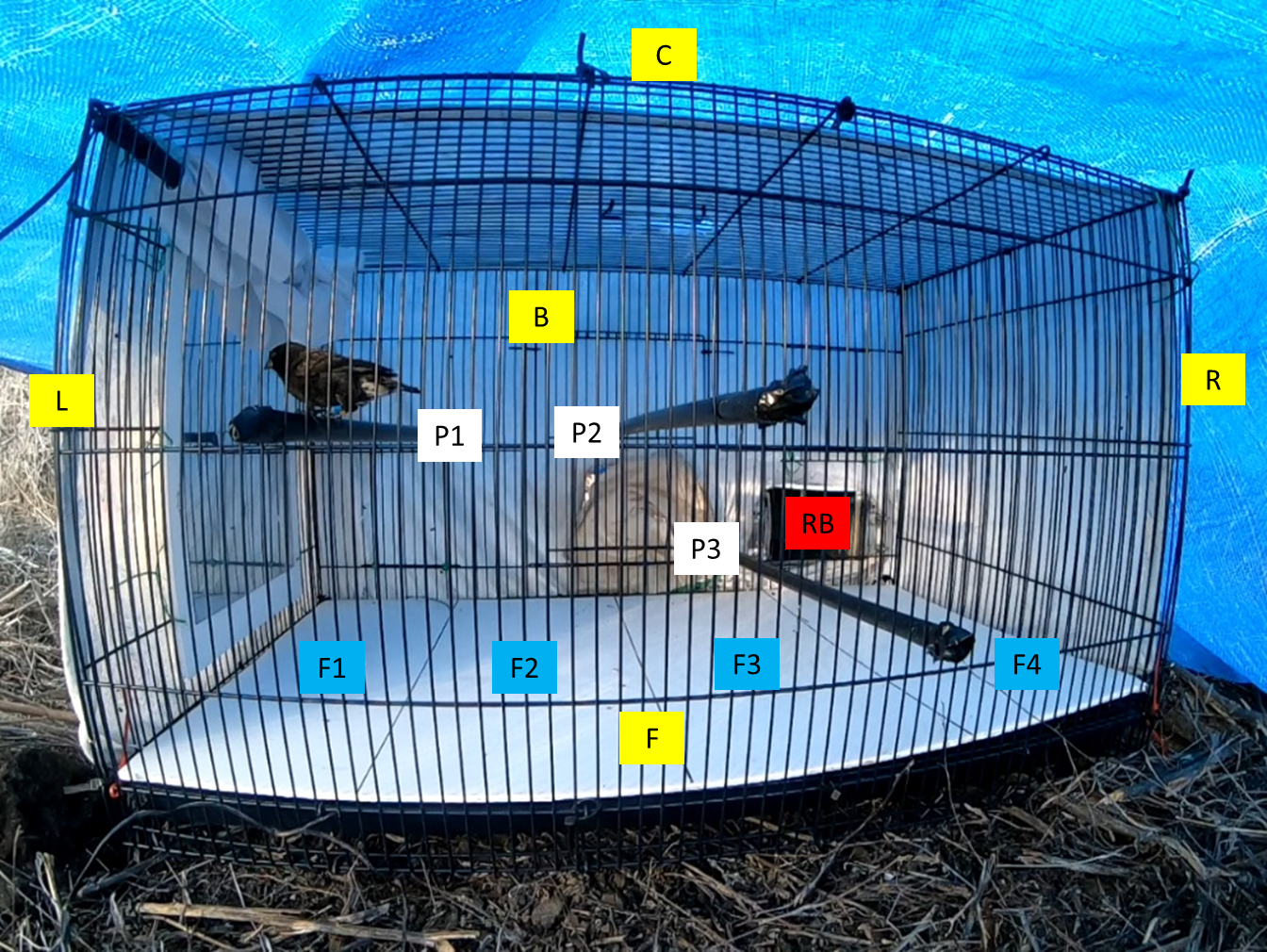


**Fig. S3** Consistency within contexts for exploration variables scored during a novel environment test (*unique sectors* and *total sectors*). The two exploration variables were positively correlated, both when species were analyzed together or separately for medium tree finches (open circles) and small ground finches (filled triangles).


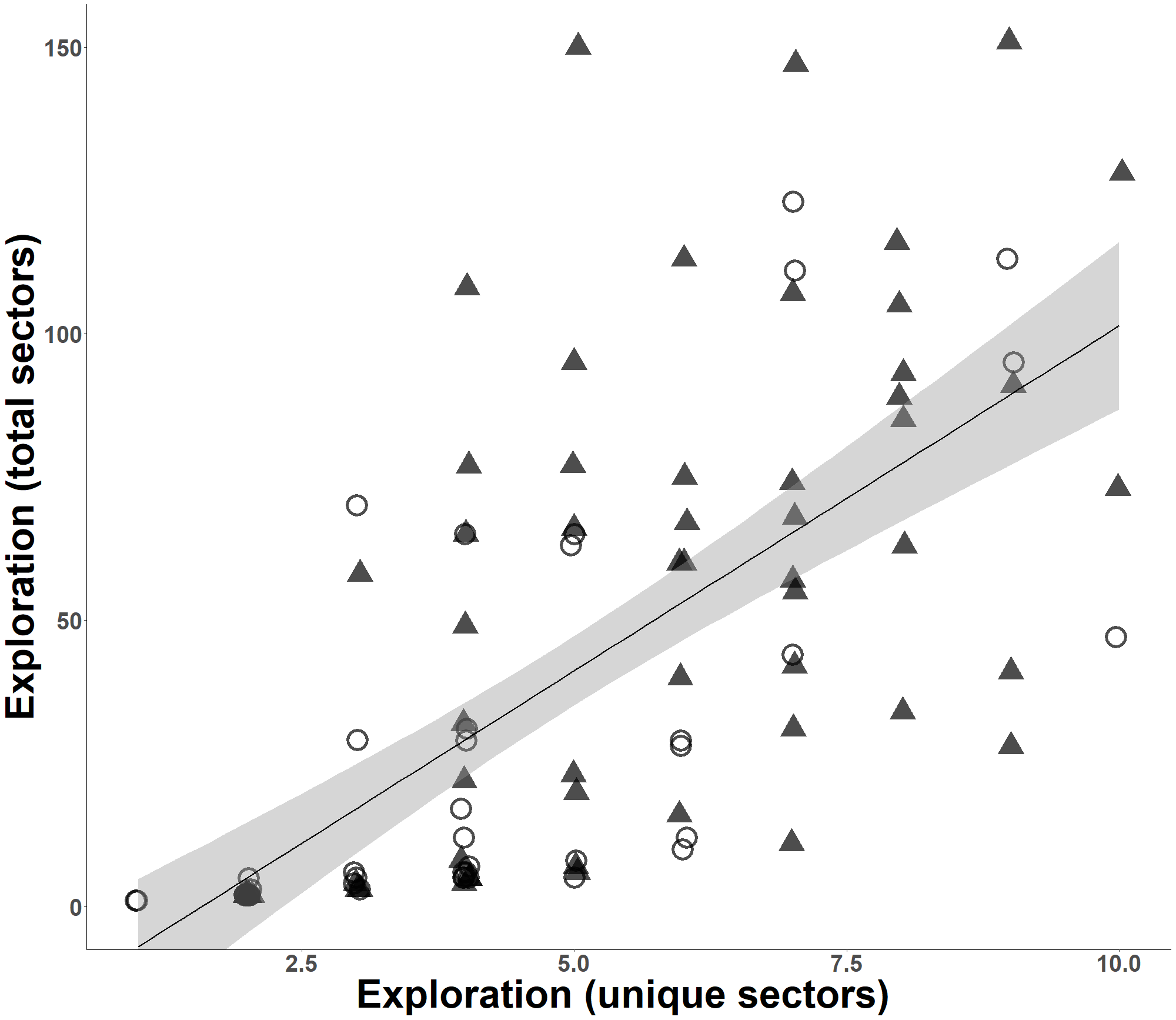


**Fig. S4** Consistency within contexts for aggressiveness variables scored using a mirror stimulation test (*time near mirror* and *mirror contact*) in medium tree finches (open circles) and small ground finches (SGF). Males that contacted the mirror (*mirror contact*) also spent significantly longer near the mirror (*time near mirror*, secs).


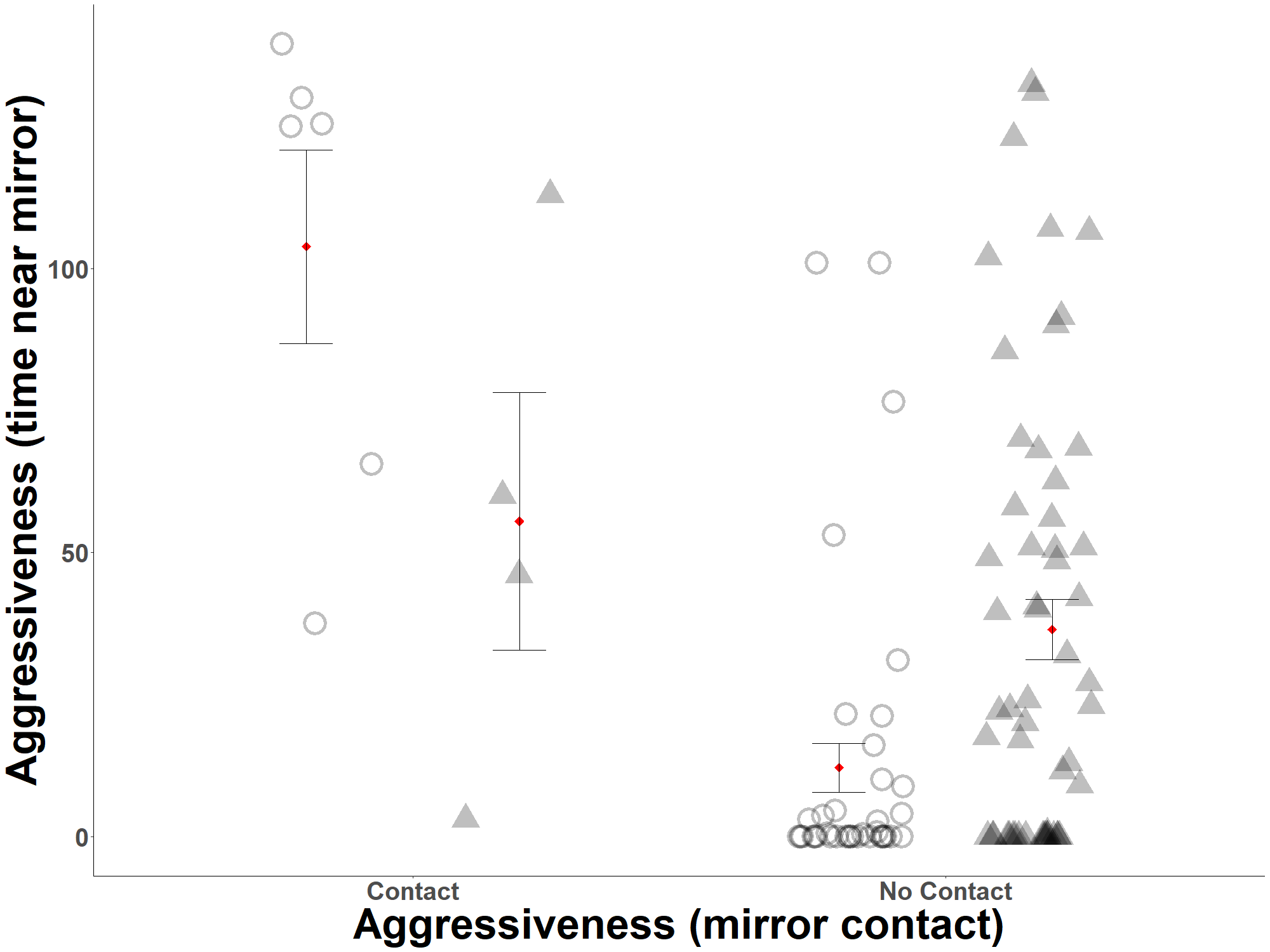


**Fig. S5** The relationship between exploration (*total sectors visited*) and log transformed home range size was not significantly associated with home range size (ha) in medium tree finches (open circles) and small ground finches (filled triangles), albeit with a negative trend (r = -0.4, *N* = 20, *p =* 0.10). The standard error level is at 95% confidence (gray shadow).


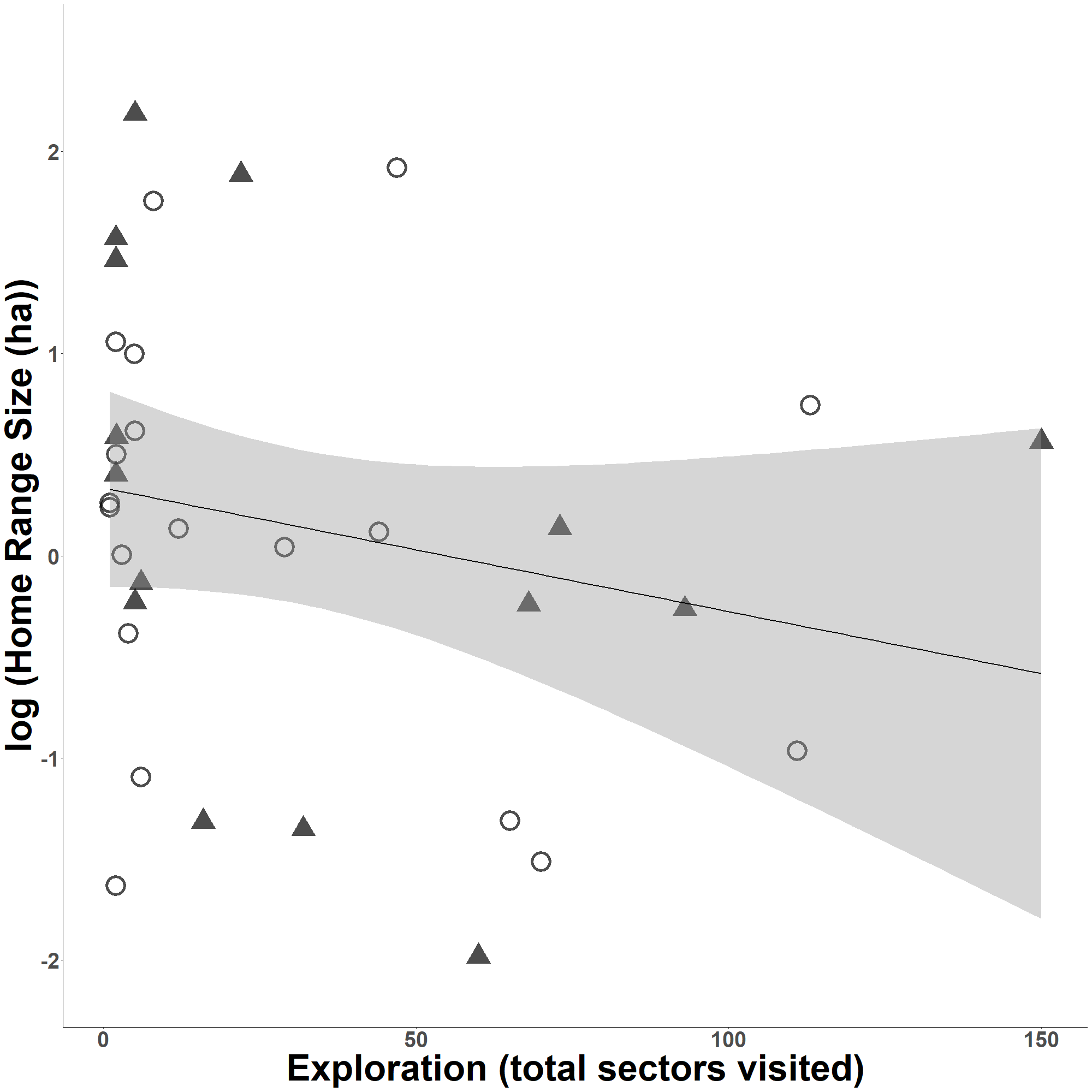


**Fig. S6** Aggressiveness in the wild (*simulated conspecific intrusions*) was not significantly associated with home range size (ha) in medium tree finches (open circles) and small ground finches (filled triangles), albeit with a negative trend (r = -0.4, *N* = 20, *p =* 0.10).


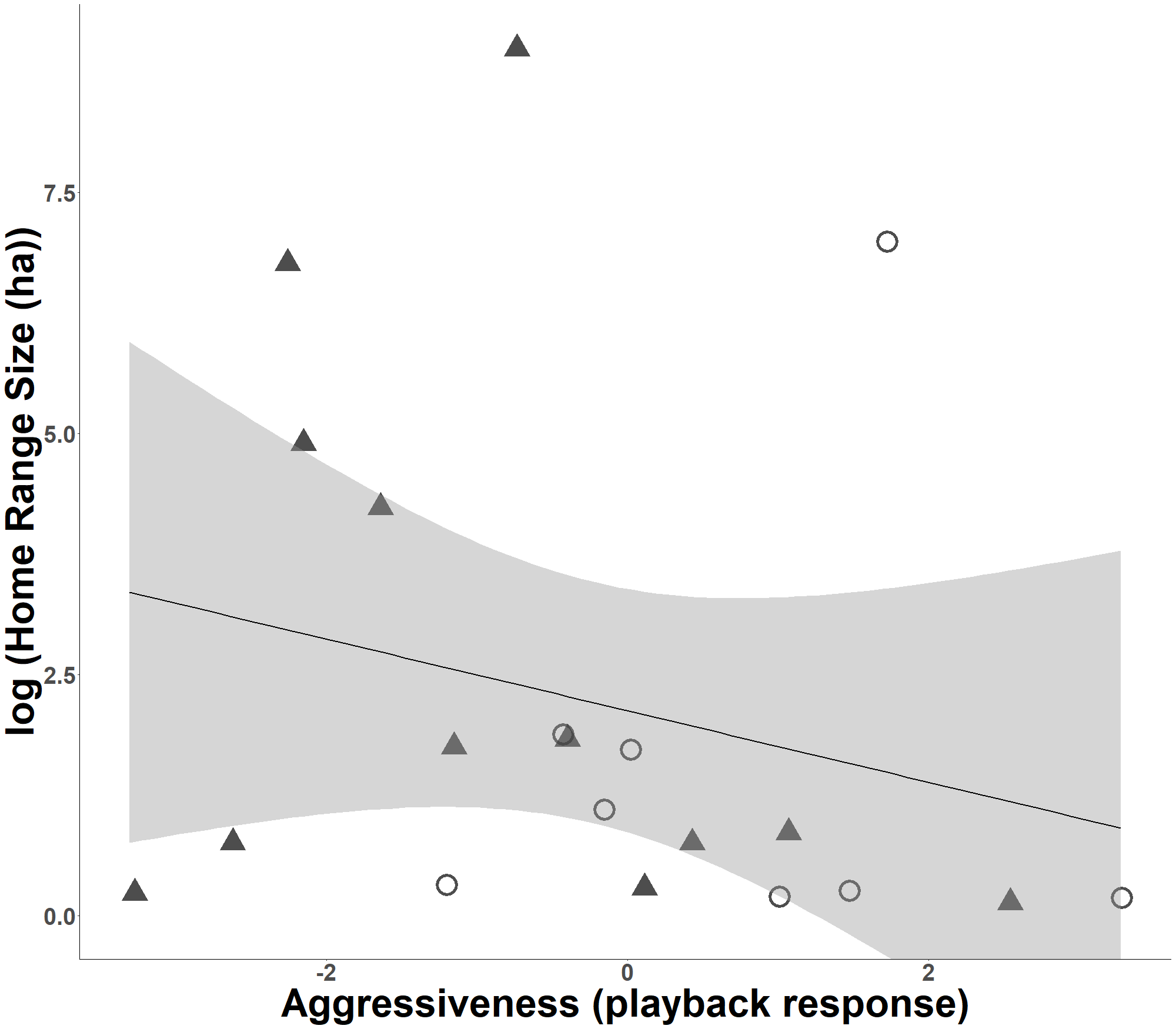


**Fig. S7** Bootstrap used to estimate the number of fixes needed, we observed how the trend starts to flattened after 13–14 fixes, which suggests that enough fixes were taken for the individuals


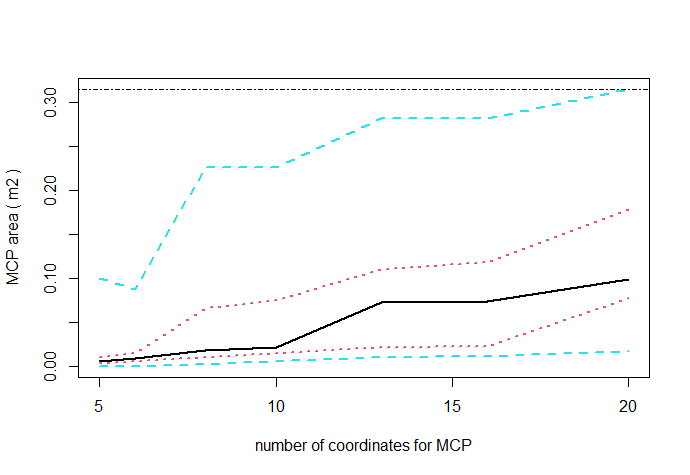

Supplement: Supplementary file 1 — Supplementary file1 (DOCX 4508 KB) [file 10336_2024_2215_MOESM1_ESM.docx]
